# Bioinformatic Analysis of Transcriptomic Data Reveals Novel Key Genes Regulating Osteogenic Differentiation of Human Adipose Stem Cells

**DOI:** 10.1155/2019/1705629

**Published:** 2019-07-31

**Authors:** Jinluo Cheng, Xinyuan Zhao, Juan Liu, Li Cui, Yanfeng Zhu, Xiaoqing Yuan, Jianbo Gao, Yunfeng Du, Xinmin Yan, Shen Hu

**Affiliations:** ^1^Changzhou Second People's Hospital, Nanjing Medical University, Changzhou, China; ^2^Stomatological Hospital, Southern Medical University, Guangzhou, China; ^3^School of Dentistry, University of California, Los Angeles, California, USA; ^4^School of Public Health, Chengdu Medical College, Chengdu, China

## Abstract

Adipose stem cells (ASCs) are an attractive cell source for treating many human diseases including osteoporosis. However, the molecular mechanisms accounting for ASC osteogenesis are poorly known. In this study, ASCs were first isolated from the fat tissues from the patients with osteoporosis. The global transcriptome profile between osteogenic differentiated ASCs and undifferentiated ASCs was compared using RNA sequencing (RNA-seq). Then, bioinformatic analysis was performed to reveal the central genes and pathways that regulated the osteogenic differentiation of ASCs. One of the interested genes C5AR1 was chosen for further investigation. A total of 1521 upregulated and 3020 downregulated genes were identified between the ASCs with osteogenic induction and controls. Functional gene ontology analysis revealed that these significantly differentially expressed genes (DEGs) were associated with cell cycle, protein binding, and nucleotide binding. Pathway analysis showed that many canonical pathways, such as the MAPK signaling pathway and the PI3K-AKT pathway, might actively be involved in regulating osteogenic differentiation of ASCs. A total of three subnetworks and 20 central nodes were identified by the protein-protein interaction analysis. In addition, the expression level of C5AR1 was significantly increased during osteogenic differentiation of ASCs. The downregulation of C5AR1 dramatically reduced the expression levels of osteogenic differentiation biomarkers and calcium nodule formation capacity. Collectively, we have provided a number of novel genes and pathways that might be indispensable for ASC osteogenic differentiation. Manipulating the levels of this candidate gene might contribute to the osteoporosis therapy.

## 1. Introduction

Osteoporosis is the most frequently occurring metabolic bone disease that affects many millions of people around the world. It is featured by low bone mineral density and microarchitectural deterioration of bone tissue, resulting in enhanced bone fragility and a consequent increase in fracture risk [1]. In recent years, accumulating evidence has suggested that bone tissue engineering seems a promising methodology for treating osteoporosis [2]. The cells, the growth factors, and the bone scaffolds are the basic elements for successful bone tissue engineering [3].

Bone marrow-derived mesenchymal stem cells (BMMSCs) are the most well-known and well-characterized source of adult stem cells [4]. In addition, BMMSC-based therapies have been widely used for treating many diseases such as cardiovascular diseases, neurodegenerative diseases, and bone defects [5, 6]. The use of BMMSCs is not always acceptable due to its several disadvantages. Firstly, obtaining BMMSCs from the bone marrow is a painful and invasive procedure and potentially exposes the donors to virus infection. Secondly, the amount of cell yield from the bone marrow is extremely minimal. Moreover, the proliferation and multipotent differentiation capacity of BMMSCs decrease with increasing senescence. Therefore, exploring a substitute cell for BMMSCs for cellular therapy is urgently needed. Adipose stem cells (ASCs) are a relatively new source of mesenchymal stem cells. They have been shown to be differentiated into cells of ectodermal, endodermal, and mesodermal origin [7–9]. In addition, the human adipose tissue is easily obtainable in large quantities with little donor site morbidity or patient discomfort [8]. Moreover, a greater number of MSCs can be yielded from adipose tissues compared to the other stem cell source [10]. These features enable them to be ideal for application in regenerative medicine.

In this study, we first aimed to determine the osteogenic potential of ASCs derived from patients with osteoporosis. Then, the key genes and pathways involved in the osteogenic differentiation of ASCs were explored by RNA sequencing and bioinformatic analysis. Finally, the role of interested gene C5AR1 in regulating ASC osteogenic differentiation was investigated.

## 2. Materials and Methods

### 2.1. Cell Culture

Human ASCs were isolated from the deep and superficial layers of abdominal fat tissues of five donors with osteoporosis and two healthy controls. All donors signed a written informed consent for using their tissues, and the study was approved by the institutional Medical Ethics Committee of Changzhou Second People's Hospital, Nanjing Medical University. Briefly, the adipose tissues were rinsed three times with sterile PBS to remove debris and red blood cells. Then, they were minced into small pieces (1-2 mm^3^) with surgical scissors. The small tissue pieces were digested in PBS containing 3 mg/mL collagenase type I (Sigma, St. Louis, MO, USA) at 37°C for 1 h. Single cell suspensions were obtained by passing the cells through a 70 *μ*m strainer (BD Falcon, San Jose, CA, USA). The ASCs were obtained by subsequent colonial expansion and cultured in the Dulbecco's modified Eagle's medium F12 supplemented with 10% fetal bovine serum, penicillin (100 U/mL), and streptomycin (100 *μ*g/mL). The cells were maintained at 37°C in an incubator containing 5% CO_2_. ASCs at passages 3 were used for the studies described herein.

### 2.2. RNA-Seq Analysis

Total RNA was extracted with the RNeasy plus mini kit (QIAGEN, Hilden, Germany) according to the manufacturer's protocol. The RNA sequencing of control and osteogenic differentiated ASCs (each with 3 biological replicates) was performed using the BGISEQ-500 sequencing system by Beijing Genomics Institute (BGI), China. Briefly, the RNA was sheared and reverse transcribed to cDNA using random primers to obtain cDNA library. Raw sequencing reads were filtered to get clean reads. Bowtie2 and HISAT were used to map clean reads to reference gene and genome, respectively. Gene expression was quantified and normalized using the RSEM tool. The NOISeq method was used to screen out differentially expressed genes (DEGs) between two groups. The standard for the cutoff value was *P* < 0.05 and absolute fold change ≥ 2.

### 2.3. Bioinformatic Analysis

Gene ontology (GO) enrichment analysis and KEGG (Kyoto Encyclopedia of Genes and Genomes) pathway analysis were performed using the Cytoscape Bingo plugin and KEGG pathway database (http://www.genome.jp/kegg/), respectively. For the protein-protein interaction (PPI) network construction, STRING online database (http://string-db.org) and Cytoscape software (Version 3.4.0, Institute for Systems Biology, Seattle, WA, http://www.cytoscape.org/) were used to identify key candidate genes that regulate the osteogenic differentiation of ASCs. Cytoscape MCODE plugin was used for searching clustered subnetworks. The default parameters were as follows: degree cutoff ≥ 2, node score cutoff ≥ 0.2, K‐core ≥ 2, and max depth = 100.

### 2.4. siRNA Knockdown of C5AR1

ASCs were transfected with double-stranded siRNA using the RNAiMAX transfection reagent (Invitrogen, Carlsbad, CA, USA) according to the manufacturer's instruction. siRNAs of C5AR1 (siC5AR1) or scrambled control siRNAs (sicontrol) (Santa Cruz Biotechnology Inc., Dallas, TX, USA) were mixed with the transfection reagent, respectively, and then added to the cell culture.

### 2.5. Real-Time PCR

Total RNA was isolated from cells sing Quick-RNA Miniprep (Zymo Research, Irvine, CA, USA) based on the manufacturer's instructions. SuperScript III Reverse Transcriptase (Invitrogen, Carlsbad, CA, USA) was used to synthesize the first-strand cDNA. The PCR reaction was performed with Light Cycler 480@ SYBR Green I Master Mix (Roche Applied Science, Indianapolis, IN, USA) on the CFX96 Real-Time PCR detection system (Bio-Rad Laboratories Inc., Hercules, CA, USA). GAPDH was employed as the internal control, and the 2^-*ΔΔ*Ct^ method was used to determine the relative expression level of target genes. Triplicate reactions were conducted in three separate experiments.

### 2.6. MTT Assay

After 24 h of serum starvation, the cells were seeded into a 96-well plate at a density of 3000 cells/well. At the indicated time points, 20 *μ*L of MTT (5 mg/mL, Sigma-Aldrich, St. Louis, MO, USA) was added to each well. Following by incubation for 4 h at 37°C, the supernatant was then discarded and the precipitate was dissolved with 200 *μ*L of dimethyl sulfoxide. The absorbance was measured using a Synergy HT microplate reader (BioTek Instruments, Winooski, VT, USA) at 570 nm.

### 2.7. Western Blotting

The proteins extracted from the cell samples were separated on a 4%-12% Bis-Tris NuPAGE gel and transferred onto a polyvinylidene difluoride membrane using a Trans-blot SD semidry transfer cell (Bio-Rad, Hercules, CA, USA). The membranes were blocked with 5% nonfat milk for 1 h at room temperature and incubated with primary C5AR1 antibody (1 : 100; Santa Cruz Biotechnology) overnight, followed by horseradish peroxidase- (HRP-) linked secondary antibody (1 : 5000; GE Healthcare, Piscataway, NJ, USA). Signal detection was performed with the ECL-Plus western blotting reagent kit (GE Healthcare).

### 2.8. Alizarin Red S Staining

Human ASCs were seeded in 6-well plates at a density of 1 × 10^5^ cells/well and grown until a confluent monolayer had formed. Osteogenic differentiation was induced by culturing them in Human Mesenchymal Stem Cell Osteogenic Differentiation Medium (Cyagen Biosciences Inc., Guangzhou, China). The medium was changed every 3 days for 3 weeks. The induced cells were fixed in ice-cold 95% ethanol for 20 min at 4°C and then stained with 2% alizarin red S for 10 min.

### 2.9. Statistical Analysis

The data were expressed as the mean ± standard deviation and analyzed by the independent sample *t*-test using the GraphPad Prism 7 software (GraphPad Software Inc., La Jolla, CA, USA). *P* value less than 0.05 was considered statistically significant.

## 3. Results

### 3.1. The DEGs between Osteogenic Differentiated ASCs and ASCs

**Figure 1:**
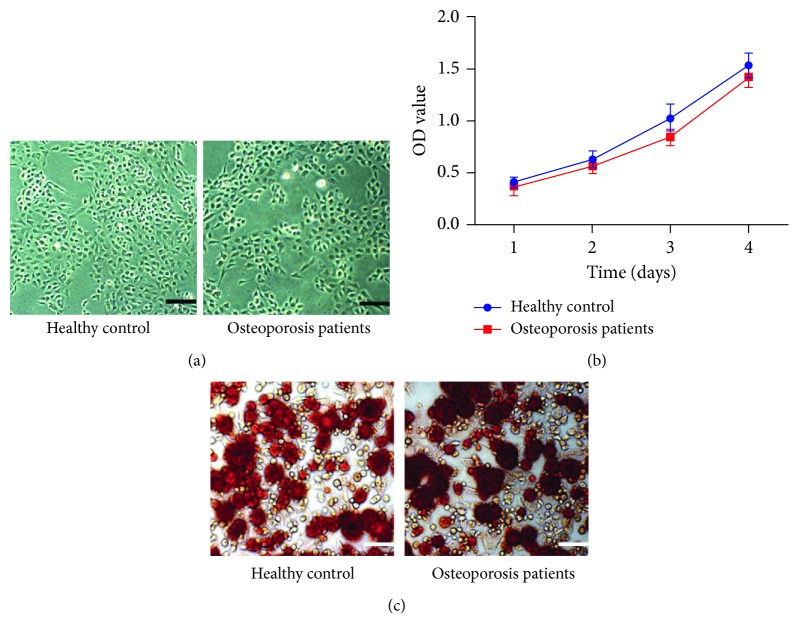
The ASCs derived from the patients with osteoporosis mainly had round-up or spindle-shaped morphology, which were similar to the ASCs from healthy donors (a). MTT assay and alizarin red S staining showed that ASCs derived from the osteoporosis patients had similar proliferation and osteogenic differentiation capacity with those from healthy controls (b, c).

**Figure 2:**
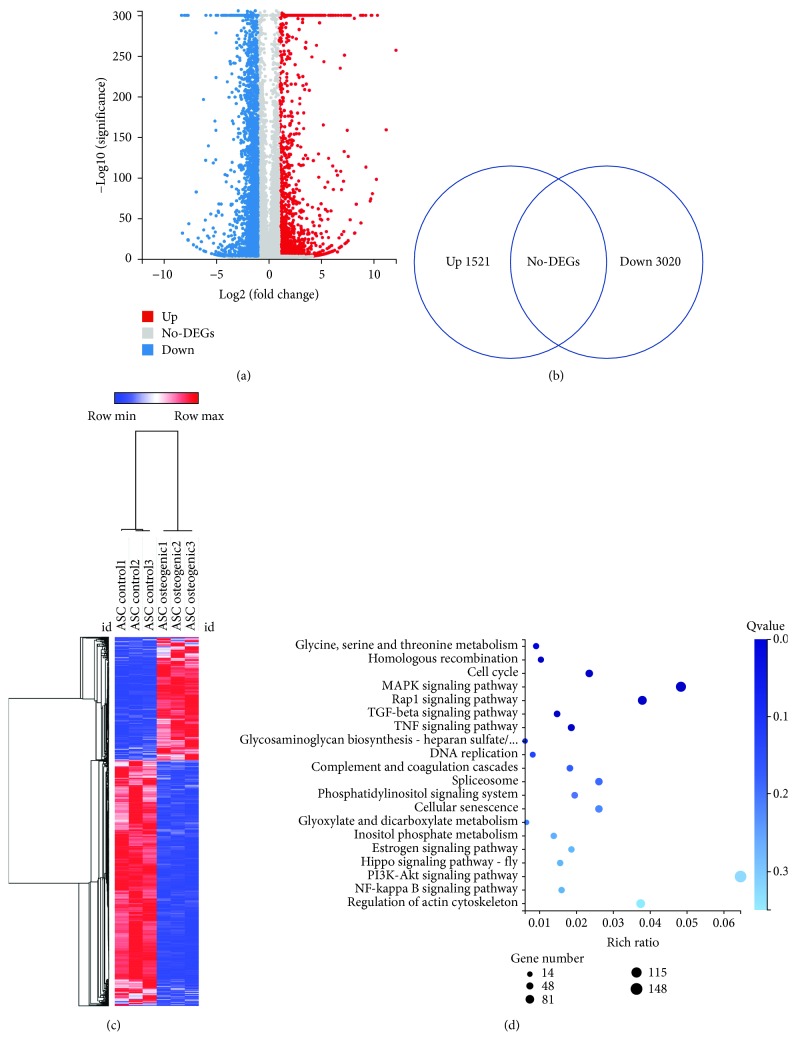
Global transcriptome profile of osteogenic differentiated ASCs and undifferentiated ASCs. (a) Volcano plot of the DEGs between osteogenic differentiated ASCs and undifferentiated ASCs. Totally, 1521 upregulated and 3020 downregulated genes were identified. (b) Heat map of the DEGs between osteogenic differentiated ASCs and undifferentiated ASCs. (c) KEGG pathway analysis of the DEGs between osteogenic differentiated ASCs and undifferentiated ASCs.

The ASCs derived from the patients with osteoporosis mainly had round-up or spindle-shaped morphology, which were similar to the ASCs from healthy donors (Figure 1(a)). In addition, they had similar proliferation and osteogenic differentiation capability compared to the ASCs from heathy donors (Figures 1(b) and 1(c)).  Figure 2(a) shows the volcano plot of DEGs that were significantly different between osteogenic differentiated ASCs and the undifferentiated controls. Each symbol represents a different gene. The red, blue, and grey color symbol indicated significantly upregulated, downregulated, and unchanged genes, respectively. Totally, 1521 upregulated and 3020 downregulated genes were identified (Supplementary data (available here)).  Figure 2(b) depicts the heat maps of DEGs between osteogenic differentiated ASCs and controls.

### 3.2. Bioinformatic Analysis of the DEGs

**Figure 3:**
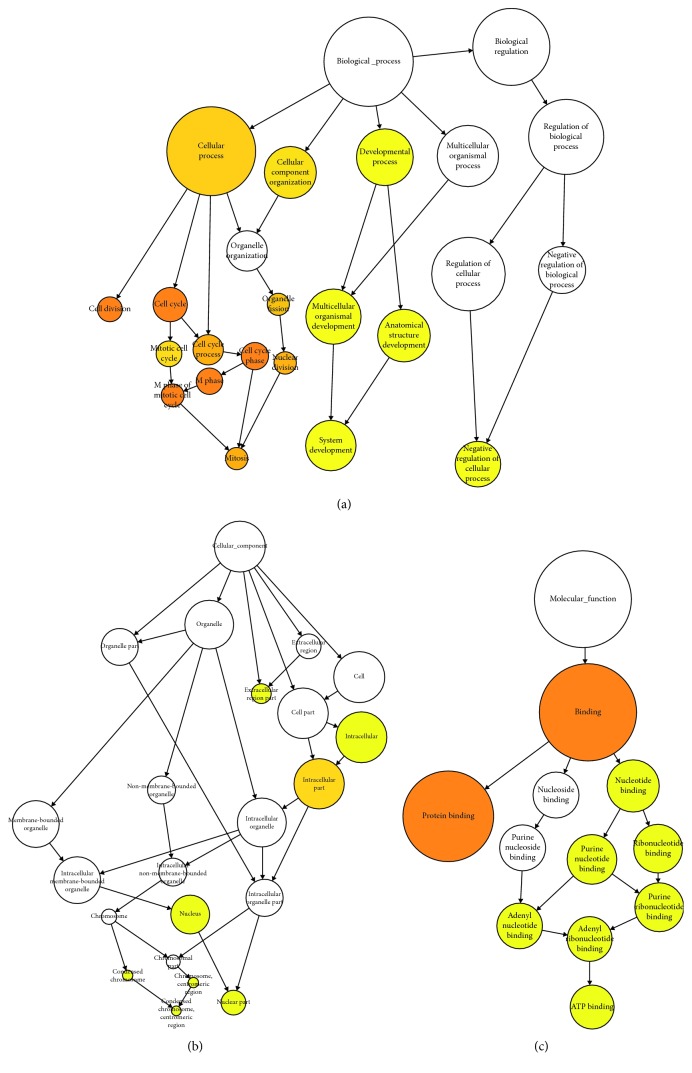
Gene ontology analyses of the significantly changed genes between osteogenic differentiated ASCs and the control cells according to their biological process, cellular component, and molecular function.

The KEGG analysis showed that the top enriched pathways included the PI3K signaling pathway, MAPK signaling pathway, cell cycle, regulation of actin cytoskeleton, and Rap1 signaling pathway (Figure 2(c)). The top biological process (BP) included cellular process, cellular component organization, cell cycle, and developmental process. The intracellular part, extracellular region part, and nucleus part are the major enriched cellular component (CC). Various binding processes such as protein binding and nucleotide binding were the top molecular function (MF) (Figure 3).

**Figure 4:**
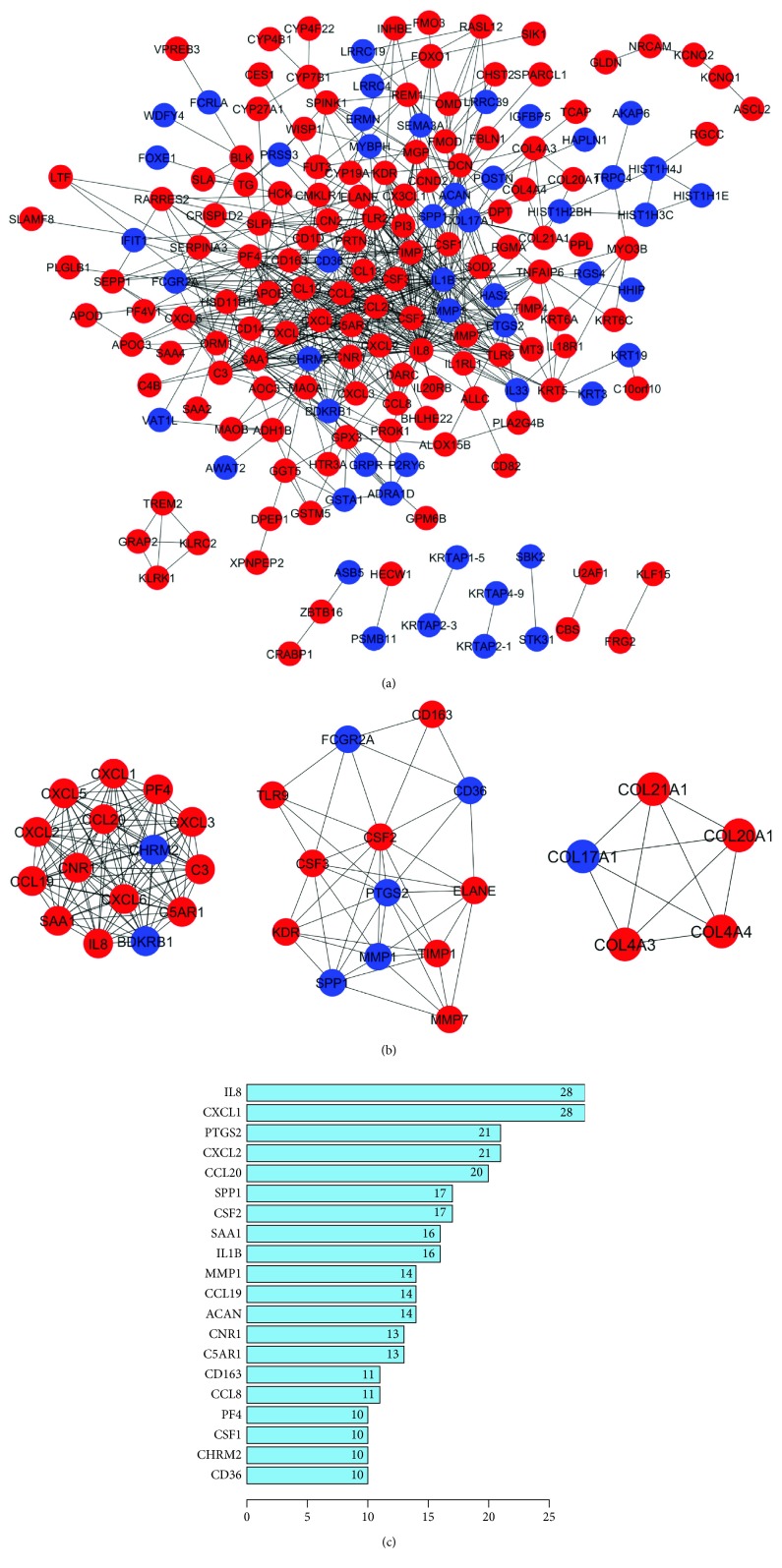
PPI network analysis of the most significant changed genes was constructed. A total of 3 subnetworks and 20 central nodes were identified by Cytoscape MCODE plugin.

Figure 4(a) shows the protein-protein interaction (PPI) network complex of the most upregulated or downregulated genes (absolute log_2_ (fold change) ≥ 4). A total of 3 subnetworks (Figure 4(b)) and 20 central nodes (connection ≥ 10) (Figure 4(c)) were identified by Cytoscape MCODE plugin. The central node genes included IL-8, CXCL1, PTGS2, CXCL2, CCL20, SPP1, CSF2, SAA1, IL1B, MMP1, CCL19, ACAN, CNR1, C5AR1, CD163, CCL8, PF4, CSF1, CHRM2, and CD36.

### 3.3. Validation of the RNA Sequencing Data

**Figure 5:**
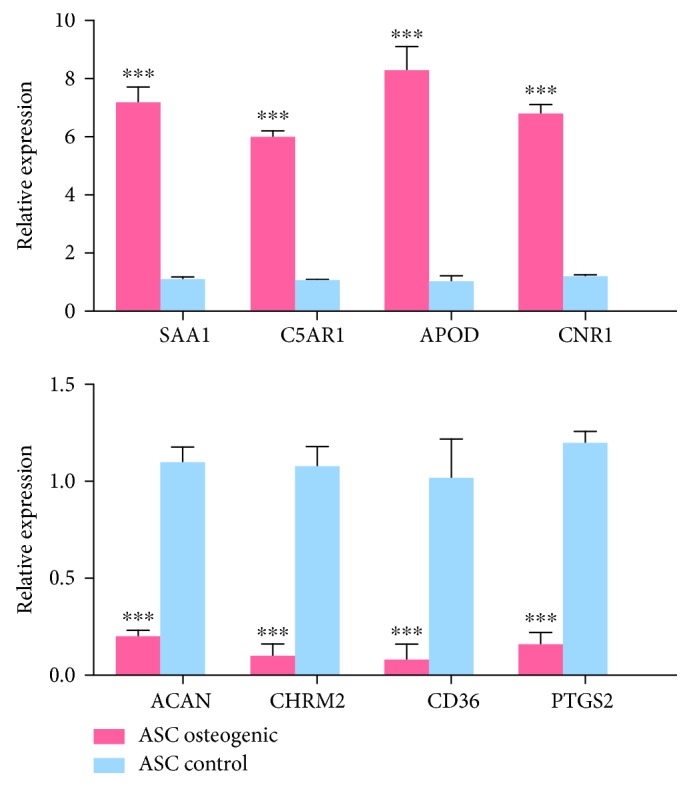
qPCR validation of the RNA-seq data. Consistent with our RNA-seq data, the expression levels of SAA1, C5AR1, APOD, and CNR1 were significantly upregulated in osteogenic differentiated ASCs, while the levels of ACAN, CHRM2, CD36, and CHRM2 were remarkably downregulated.

Our qPCR data showed that the expression levels of SAA1, C5AR1, APOD, and CNR1 were significantly increased in osteogenic differentiated ASCs compared with the undifferentiated controls. On the contrary, the expression levels of ACAN, CHRM2, CD36, and CHRM2 were dramatically reduced in ASCs with osteogenic induction (Figure 5). These results were consistent with our transcriptome data.

### 3.4. Suppression of C5AR1 Inhibited the Osteogenic Differentiation of ASCs

**Figure 6:**
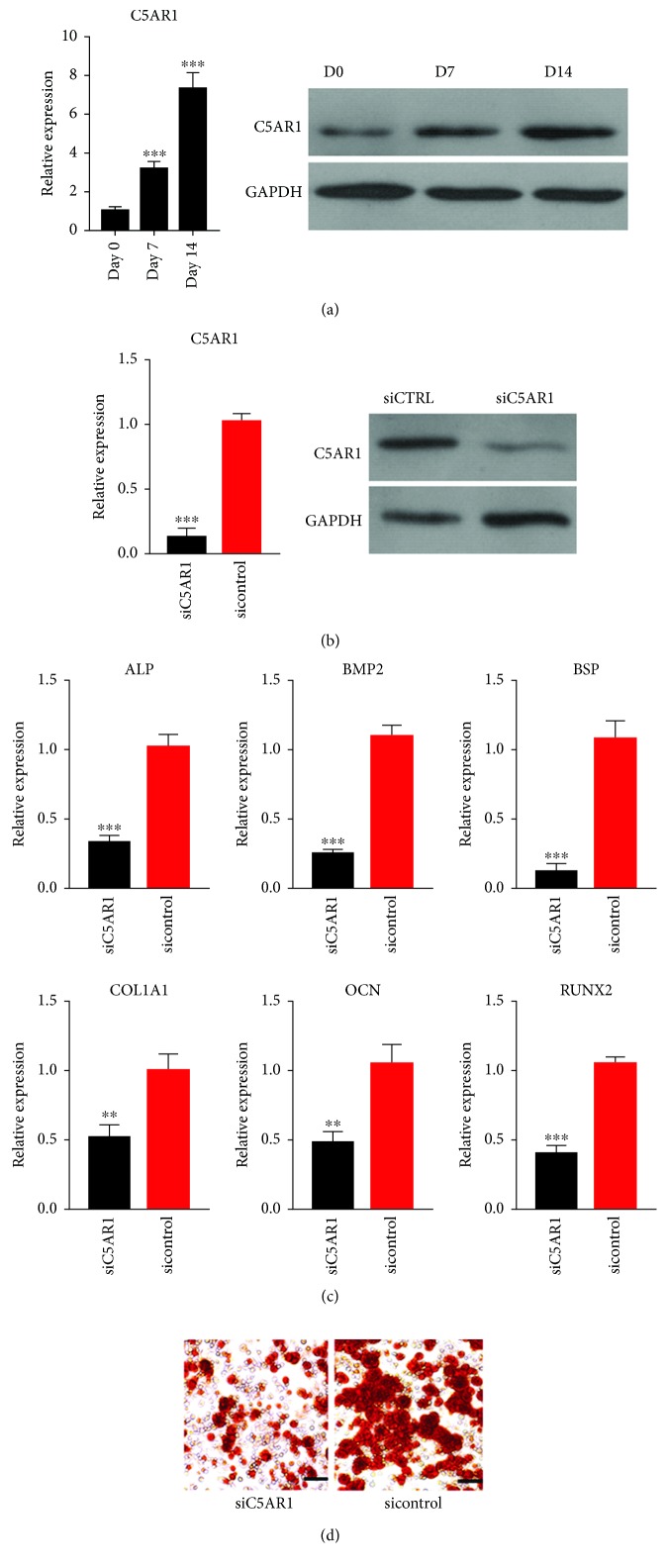
The inhibition of C5AR1 suppressed the osteogenic differentiation of ASCs. (a) The expression of C5AR1 mRNA and protein was significantly increased during osteogenic differentiation of ASCs. (b) The expression level of C5AR1 mRNA and protein was dramatically downregulated following siC5AR1 transfection. (c) C5AR1 downregulation inhibited the expression of osteogenic differentiation markers. (d) C5AR1 downregulation suppressed the calcium nodule formation capacity of ASCs.

The expression levels of C5AR1 mRNA and protein were gradually increased with osteogenic induction (Figure 6(a)). The C5AR1 siRNA we transfected could significantly suppress the expression level of C5AR1 at both mRNA and protein levels in ASCs (Figure 6(b)). Following a two-week osteogenic induction, the expression levels of osteogenic markers (ALP, BMP2, BSP, RUNX2, OCN, and COL1A1) were remarkably lower in the C5AR1 knockdown group compared with the control siRNA group (Figure 6(c)). In addition, alizarin red S staining revealed that the number of calcium nodules was significantly lower in the siC5AR1 group compared with the control group (Figure 6(d)).

## 4. Discussion

In this study, we have shown that the ASCs derived from patients with osteoporosis had strong osteogenic differentiation potential, which further supports the usage of ASCs as the seed cells for treating osteoporosis to avoid the potential graft rejection owing to allograft. In addition, many significantly upregulated or downregulated genes during osteogenic differentiation of ASCs were identified. Bioinformatic analysis revealed many key central genes and pathways that might play essential roles in regulating ASC osteogenic differentiation. To the best of our knowledge, many genes are reported to be potential regulators of ASC osteogenic differentiation for the first time. Moreover, the expression level of the central node gene C5AR1 was gradually increased during osteogenic differentiation of ASCs. The downregulation of C5AR1 significantly suppressed the osteogenic differentiation of ASCs, indicating that C5AR1 was a positive regulator of ASC osteogenic differentiation.

The KEGG pathway analysis revealed many important pathways that might be involved in regulating osteogenic differentiation of ASCs. For instance, the MAPK signaling pathway has been demonstrated to function as key players in skeletal development and bone homeostasis especially osteoblast commitment and differentiation [11]. Also, the activation of the PI3K-AKT signaling pathway was required for the osteogenesis of human MSCs [12]. Cell cycle was the top enriched biological process during osteogenic differentiation of ASCs. The association between cell proliferation and differentiation is complex and characterized by mutual dependencies [13]. For the cellular components, all the components such as the intracellular part, nucleus, and extracellular region were enriched, suggesting that the cellular organelles in these parts were actively involved in ASC osteogenic differentiation. Protein binding and nucleotide binding were the top enriched molecular function. Nucleotide-binding proteins are essential for a number of pivotal cellular processes including, but not limited to, cell signaling, proliferation, differentiation, and survival [14].

We then used the most upregulated and downregulated genes to construct the PPI network. Our results identified many interested genes that might play an important role in regulating osteogenic differentiation of ASCs. For instance, recombined serum amyloid A (SAA) could induce the osteogenic differentiation of MSCs by regulating the TLR4 receptor [15], which supported our findings that the expression level of SAA was dramatically increased in ASCs with osteogenic induction. SPP1, namely, osteopontin (OPN), is a well-recognized osteogenic differentiation biomarker [16]. Its upregulation in osteogenic differentiated ASCs further supports our RNA-seq data. In addition, many genes from the chemokine CXC or CC chemokine family such as CXCL1, CXCL2, CCL8, CCL19, and CCL20 were found to be upregulated during the osteogenic differentiation of ASCs. CXC chemokines are crucial and essential for osteogenic differentiation of BMMSCs [17]. Further studies are warranted to explore the role of chemokines in ASC osteogenesis.

C5AR1 is a G protein-coupled receptor for C5a and also an N-linked glycosylated protein [18]. C5A/C5AR1 axis plays an important role in regulating innate immunity and inflammation process [19, 20]. The expression level of C5AR1 was gradually increased during the osteogenic differentiation of ASCs, indicating that C5AR1 might be actively involved in the differentiation process. We further demonstrated that specific downregulation of C5AR1 significantly reduced the osteogenic potential of ASCs, suggesting that C5AR1 might be a crucial regulator which controls the osteogenic property of ASCs. However, it seemed that the knockdown of C5AR1 in ASCs caused little changes in morphology and the proliferation capacity. Consistent with our findings, the expression level of C5AR1 was also gradually increased during the osteogenic differentiation of human BMMSCs. In addition, C5AR1 might be important for fracture healing in the ossification process [21]. Similarly, Kalbasi Anaraki et al. reported that C5AR1 was upregulated upon differentiation of human BMMSCs to osteoblasts. The inhibition of C5AR1 suppressed the osteogenic differentiation of human BMMSCs, and this process might be mediated by the urokinase receptor [22]. C5AR1 is important for the osteogenic differentiation of MSCs derived from different sources, indicating that it might play a conserved role in regulating the osteogenic process. However, the underlying molecular mechanisms accounting for the role of C5AR1 in regulating osteogenic differentiation of ASCs need further investigation.

## 5. Conclusion

In conclusion, based on our reliable transcriptome data, we have identified many novel genes and pathways that might be involved in regulating the osteogenic differentiation of ASCs. In addition, C5AR1 might act as a positive modulator for ASC osteogenic differentiation, indicating that manipulating the C5AR1 expression level might contribute to bone regeneration and tissue engineering.

## Data Availability

The RNA-seq data used to support the findings of this study are included within the supplementary information file.

## Supplementary Materials

Supplementary Materials The significant differentially expressed genes between osteogenic differentiated ASCs and controls.
